# Effectiveness of the Combination of Rituximab and Standard Chemotherapeutic Regimens in Previously Untreated Patients with Chronic Lymphocytic Leukaemia in Real-Life: Results from a Noninterventional Study (CILI Study)

**DOI:** 10.1007/s12253-018-0474-9

**Published:** 2018-10-25

**Authors:** Róbert Szász, Elvira Altai, Katalin Pál, Péter Dombi, János Iványi, János Jakucs, Natália Jóni, Árpád Illés, Ilona Tárkányi, László Szerafin, Zsolt Nagy, Péter Farkas, Ágnes Nagy, Klára Piukovics, György Ujj, Tamás Schneider

**Affiliations:** 1 Division of Hematology, Department of Internal Medicine, Faculty of Medicine, University of Debrecen, Nagyerdei krt. 98, Debrecen, 4032 Hungary; 2 Veszprém County Csolnoky Ferenc Hospital, Kórház u. 1, Veszprém, 8200 Hungary; 3 Fejér County Szent György Hospital, Seregélyesi út 3, Székesfehérvár, 8000 Hungary; 4 Department of Hematology, Szent Borbála Hospital, Dózsa György út 77, Tatabánya, 2800 Hungary; 5 Department of Hematology, Vas County Markusovszky Hospital, Markusovszky L. u. 5, Szombathely, 9700 Hungary; 6 Department of Internal Medicine, Békés County Pándy Kálmán Hospital, I, Semmelweis u. 1, Gyula, 5700 Hungary; 7 Markhot Ferenc Hospital, Széchenyi u. 27-29, Eger, 3300 Hungary; 8 1st Department of Internal Medicine, Semmelweis University, Korányi Sándor u. 2/A, Budapest, 1083 Hungary; 9 Department of Haematology, Szabolcs-Szatmár-Bereg County Jósa András Hospital, Szent István u. 68, Nyíregyháza, 4400 Hungary; 10 II. Department of Internal Medicine, Semmelweis Hospital, Csabai kapu 9-11, Miskolc, 3529 Hungary; 11 3rd Department of Internal Medicine, Semmelweis University, Kútvölgyi út 4, Budapest, 1125 Hungary; 12 I. Department of Internal Medicine, University of Pécs, Ifjúság út 13, Pécs, 7624 Hungary; 13 II. Department of Internal Medicine, University of Szeged, Korányi Fasor 6, Szeged, 6720 Hungary; 14 I. Department of Internal Medicine, Jász-Nagykun-Szolnok County Hetényi Géza Hospital, Tószegi út 21, Szolnok, 5004 Hungary; 15 “A” Department of Internal Medicine, National Institute of Oncology, Ráth György u. 7-9, Budapest, 1122 Hungary

**Keywords:** Chronic lymphocytic leukemia, Rituximab, Overall response rate

## Abstract

Chronic lymphocytic leukemia (CLL) is one of the most common haematological malignancies exhibiting remarkable heterogeneity in clinical course. Rituximab added to standard chemotherapy has been proven to increase response rate and eventually survival among previously untreated CLL patients. CILI was an open-label, non-randomized, single arm, multicentric, observational study aimed to collect real-life effectiveness data for rituximab used according to the current label in combination with standard chemotherapy in previously untreated CLL patients. Overall response rates (ORR) in the entire study population as well as in various subgroups were estimated. Adverse events were recorded during the entire course of the study. A total number of 150 patients were enrolled by 15 Hungarian study sites. Out of these, 82 patients received 6 cycles of rituximab containing treatment. Overall response rates of 88.24% (CI_95%_: 81.6–93.12%) and 94.59% (CI_95%_: 86.73–98.51%) were recorded in the intent-to-treat (ITT) and per-protocol (PP) populations, respectively. In both study populations, somewhat higher ORR was observed in patients aged ≥65 years. Subgroups defined according to either chromosomal aberrations (presence of 11q and 17p deletions) showed apparently high ORRs, though these rates were most probably biased by low patient numbers. 144 adverse events were reported during the study, of which 15 AEs were considered to be related to the administration of rituximab. Analyses of the efficacy variables have revealed comparable results to those previously reported by controlled clinical trials.

## Introduction

Chronic lymphocytic leukemia (CLL) is a malignant lymphoproliferative disease characterized by uncontrolled replication of B-lymphocytes. CLL is the most common subtype of leukemia in the Western world with an incidence of 4.2 cases per 100,000 persons per year [1]. CLL affects mostly elderly patients; the median age at the time of diagnosis was reported to be 70 years. Familial predisposition is presumable, since 5%–10% of the patients with CLL have a family history of lymphoid malignancies [1]. CD19 positive CLL B-cells are characterized by the increased expression of CD5 and CD23 antigens and the low expression of CD20, CD79b, and surface immunoglobulin. Blood smear morphology typically shows mature lymphocytes and Gumprecht shadows [2].

The clinical course of CLL exhibits remarkable heterogeneity as some patients may have a normal life expectancy without requiring treatment while others experience rapidly progressing disease [3]. Considerable efforts have been directed towards the development of prognostic indices for the stratification of patients into subgroups with distinct clinical outcome. Amongst these, clinical staging systems introduced by Binet et al. [3] and Rai et al. [4] are the most widely accepted in the clinical practice, though these fail to identify patients in early clinical stage who will experience an aggressive disease course [5].

During the last few decades, several biomarkers and other factors have been reported that correlate with the probability of disease progression at the time of diagnosis. Of these, somatic hypermutation status of the immunoglobulin heavy variable (IGHV) genes, cytogenetic abnormalities (most prominently 11q and 17p deletions) and TP53 mutation are of particular importance [5]. Patients with 11q and 17p deletions/TP53 mutation had intermediate and poor prognosis, respectively, as reflected from significantly lower 5-year progression free survival. Moreover, 11q deletion is associated with marked lymphadenopathy and rapid disease progression, while 17p deletion/TP53 mutation is predictive of treatment failure with alkylating agents and fludarabine [6].

Provided that no survival benefit is associated with early chemoimmunotherapy, treatment is usually initiated when patients become symptomatic or progress to late stage. In the last decades, first-line treatment of CLL has evolved from single-agent therapy with alkylating drugs (e.g., chlorambucil) to combination therapy incorporating purine analogues (i.e. fludarabine, pentostatin and cladribine) and monoclonal antibodies (i.e., rituximab and obinutuzumab) [7–12].

Rituximab is a chimeric murine/human monoclonal antibody that is directed specifically against the B cell antigen CD20 [13]. Rituximab was shown to induce both complement-mediated and antibody-dependent cell-mediated lysis of CD20+ cells [14]. Moreover, sensitization of drug-resistant human B cell lymphoma cell lines to cytotoxic agents has also been observed [15]. Clinical data showed the rapid depletion of CD20+ B-cells 24–72 h after rituximab administration. This effect was apparent even after 2–3 months of therapy [16]. Since CLL-cells show low level CD20 expression, higher doses of rituximab are required to achieve favourable therapeutic outcome.

Currently, fludarabine, cyclophosphamide, and rituximab (FCR) is the standard of care in previously untreated fit CLL patients with treatment requirement [17]. However, the favourable outcome of FCR treatment has been reported only for patients not suffering from other medical impairments. Provided that more than two thirds of the CLL patients are 65 years old or more, concurrent pathological conditions and/or physiological decline of organ function are commonly present. Relevant co-morbidities have a substantial effect on the outcome of the treatment, since such patients have a higher risk to develop side-effects and thus require alternative, less toxic therapies [18]. Today, anti-CD20 therapy (rituximab or obinutuzumab) in combination with chlorambucil is recommended for patients with significant comorbidities and rituximab plus bendamustin for older fit patients [19, 20].

This non-interventional study was designed to collect real-life effectiveness data for rituximab used in combination with standard chemotherapy in previously untreated CLL patients. In addition, it was aimed to evaluate of the usage of different concomitant chemotherapeutic regimens in the routine clinical practice as well as the effect of different prognostic factors on response rates. CILI is a word-play between CLL and a common pet name, not an acronym. The protocol was prepared in 2013 and the data collection period was between 26 April 2014 and 19 Dec 2016.

## Materials and Methods

### Study Design and Patients

This open-label, non-randomized, single arm, multicentric, observational study was designed to collect real-life efficacy and safety data. Adult patients with medically confirmed, previously untreated chronic lymphoid leukaemia who started their first line treatment with rituximab and standard chemotherapy in line with the effective Summaries of Product Characteristics were eligible for participation. Patients were enrolled after having received the first cycle of the treatment. Pregnant and breastfeeding patients as well as those who had received any investigational medicinal products 30 days prior to enrollment were excluded from the study population. All patients were required to provide written informed consent before entering the study.

### Treatment

Patients were assessed and treated according to everyday clinical practice in line with the relevant therapeutic guidelines and protocols. No new diagnostic or therapeutic options were tested in this non-interventional study.

Eligible subjects were projected to receive 6 cycles of rituximab treatment in combination with standard chemotherapy. According to the current label, rituximab 375 mg/m^2^ body surface area administered on day 0 of the first treatment cycle followed by 500 mg/m^2^ body surface area administered on day 1 of each subsequent cycle. The choice of therapy was based exclusively on the medical decision of the treating physician before study enrollment, and medication was ordered independently of the study. Patients were enrolled in the study after having received the first cycle of the treatment.

### Procedures

After enrollment, study data were collected on five consecutive treatment visits (i.e. after administration of each cycle) and on the final visit, 2 months after the administration of the last cycle. On the first visit, treating physicians recorded the patient’s demographic data (age and sex), compliance to the inclusion and exclusion criteria, vital parameters (body surface), CLL specific medical history (Binet stage, CIRS score) and chromosomal mutation status (presence of 11p or 17q deletions). On all treatment visits (1–5 visits and final visit) data related to rituximab therapy and concomitant chemotherapy (regimen and dosage) as well as adverse events were recorded. Best response was evaluated by using routine assessment techniques and the result was recorded on the final visit.

The primary objective of the study was to evaluate the benefit of the first-line rituximab and chemotherapy in the target population. The primary efficacy variable was the overall response rate (ORR) defined as the proportion of patients showing partial or complete response after first-line treatment. The secondary objective of the study was to determine the ORR in various subgroups defined according to age (<65 yrs., ≥65 yrs), CIRS score (ranging from 0 to 22) and chromosomal mutation status (presence of 17p or 11q deletions).

### Statistical Analysis

Demographic characteristics, vital parameters, CLL-related parameters (i.e. Binet stage, CIRS score, chromosomal mutations, dosing of rituximab and chemotherapeutic regimens) as well as reported adverse events were analysed by descriptive statistical methods. Overall response rates defined as primary and secondary endpoints were characterized by using point estimates and 95% confidence intervals.

## Results

A total number of 150 (92 males and 58 females) patients (*intent-to-treat* (ITT) population) were found eligible for participation in the study as fulfilled all inclusion criteria and thus were enrolled by 15 clinical centres in Hungary. In summary, 82 patients (54.67% of the ITT population) received 6 treatment cycles, out of which 78 patients (52%, 54 males, 24 females) were included in the per-protocol (PP) population. Patients excluded from the PP population did not received 6 treatment cycles mainly because of adverse event (24 patients), death due to other reasons (5 patients), withdrawal of informed consent (5 patients), disease progression (4 patients) or other reasons (30 patients). Additionally, 4 patients were also excluded from the PP population because of protocol violation, though they received 6 treatment cycles.

In the ITT population, the median age was 68.55 years (range: 39.25–89.52 years). The proportion of patients aged ≥65 years was slightly higher (56.67%) compared to the proportion of patients aged <65 years (43.33%). Similar demographical characteristics was observed in the PP population (median age: 69.86 years, range: 39.25–88.48 years; aged <65 years: 39.74%, aged ≥65 years: 60.26%).

In both populations, most of the patients were ranked to Binet stage B (ITT: 45.33%, PP: 42.31%) and stage C (ITT: 44.00%, PP: 48.72%). CIRS scores were at or below 6 in the majority of the patients irrespective of study population indicating a relatively low rate of co-morbidities at enrollment. The mean CIRS score was slightly lower in the ITT population (4.278 ± 4.511) compared to those observed in the PP population (5.583 ± 4.986).

Chromosomal mutations relevant for CLL were observed only in a small fraction of patients. In the ITT population 17p and 11 q deletions were found in 3 (2.00%) and 8 (5.33%) patients, respectively. Of these, 2 patients with 17p deletion and 6 patients with 11q deletion were included in the PP populations as well.

A considerable heterogeneity has been observed regarding the concomitantly applied standard chemotherapy. In the ITT population, the most frequently used regimens were fludarabine/cyclophosphamide (64 patients, 42.67%), cyclophosphamide/vincristine /prednisolone (26 patients, 17.33%) and chlorambucil (21 patients, 14.00%). These three regimens were the most frequent in the PP population as well (Table 1).

**Table 1:** Distribution of study populations according to concomitant chemotherapeutic regimens

Chemotherapeutic regimen	ITT		PP	
	N	%	N	%
Chlorambucil (< 100 mg/cycle)	21	14.00%	11	14.10%
Chlorambucil (≥ 100 mg/cycle)	2	1.33%	2	2.56%
Cyclophosphamide/vincristine/prednisolone (CVP)	26	17.33%	17	21.79
Cyclophosphamide/doxorubicin/vincristine/prednisolone (Standard CHOP)	1	0.67%	1	1.28%
Cyclophosphamide/doxorubicin/vincristine/prednisolone (Modified CHOP by Binet)	3	2.00%	1	1.28%
Cyclophosphamide/doxorubicin/prednisolone (CAP)	1	0.67%	0	0%
Fludarabine	9	6.00%	5	6.41%
Fludarabine/cyclophosphamide (Standard FC)	64	42.67%	29	37.18%
Fludarabine/cyclophosphamide (dose decreased)	8	5.33%	3	3.85%
Bendamustin (90 mg/m^2^)	9	6.00%	5	6.41%
Bendamustin (70 mg/m^2^)	6	4.00%	4	5.13%
Total:	150	100%	78	100%

Analysis of the entire ITT population has revealed an ORR of 88.24% (CI_95%_: 81.6–93.12%). In comparison, the ORR in the PP population was 94.59% (CI_95%_: 86.73–98.51%).

In both study populations, somewhat higher ORR could be observed in patients aged at or above 65 years compared to those aged below 65 yrs. The lowest ORR was observed in patients of the ITT population aged below 65 years (86.54%, CI_95%_: 74.21–94.41%), whereas the highest ORR was found in the PP population in patients aged at or above 65 years (97.62%, CI_95%_: 87.43–99.94%).

In the ITT population, presence of both 17p and 11q deletions resulted in lower ORR compared to those without these mutations. The presence of 11q deletion was associated with lower ORR in the PP population as well (80% vs. 97.22%). On contrary, the negative effect of 17p deletion was unclear in the PP population as the ORR was found to be higher in patients with demonstrated presence of 17p deletion (100% vs. 95.42%).

No clear tendency between CIRS scores and corresponding ORR was observed in either study populations. In the ITT population, the lowest ORR was found among patients with CIRS score 5 (62.5%, CI_95%_: 24.49–91.48%), whereas in the PP population patients with CIRS score 1 and 6 had the lowest ORR (80%, CI_95%_: 28.36–99.49%). Irrespective of study population, subgroups with CIRS score of ≥7 showed ORR of 100% (Table 2).

**Table 2:** Overall reponse rates

	ITT population			PP population		
	N	Point estimate	CI_95%_	N	Point estimate	CI_95%_
Overall population	150	88.24%	81.6–93.12%	78	94.59%	86.73–98.51%
ORR by Age groups						
< 65 yrs	65	86.54%	74.21–94.41%	31	88%	68.78–97.45%
≥ 65 yrs	85	90.91%	81.26–96.59%	47	97.62%	87.43–99.94%
ORR by 17p deletion						
Present	3	71.43%	9.43–99.16%	2	100%	15.81–100%
Not present	87	90.77%	81.02–95.53%	48	95.42%	84.53–99.44%
No data	60	87.50%	75.1–94.63%	28	92.86%	76.5–99.12%
ORR by 11q deletion						
Present	8	71.43%	29.04–96.33%	6	80%	28.36–99.49%
Not present	72	90.77%	80.98–96.54%	39	97.22%	85.47–99.93%
No data	70	87.50%	76.85–94.45%	33	93.94%	79.77–99.26%
ORR by CIRS score						
0	20	78.95%	54.43–93.95%	6	100%	54.07–100%
1	12	81.82%	48.22–97.72%	5	80%	28.36–99.49%
2	14	91.67%	61.52–99.79%	7	85.71%	42.13–99.64%
3	12	100%	73.54–100%	8	100%	63.06–100%
4	13	91.67%	61.52–99.79%	5	100%	47.82–100%
5	10	62.50%	24.49–91.48%	6	83.33%	35.88–99.58%
6	6	83.33%	35.88–99.58%	5	80%	28.36–99.49%
7	5	100%	47.82–100%	4	100%	39.76–100%
9	2	100%	15.81–100%	2	100%	15.81–100%
10	2	100%	15.81–100%	2	100%	15.81–100%
11	3	100%	29.24–100%	3	100%	29.24–100%
12	3	100%	29.24–100%	2	100%	15.81–100%
15	1	100%	2.5–100%	1	100%	2.5–100%
16	1	100%	2.5–100%	1	100%	2.5–100%
17	1	100%	2.5–100%	0	0%	–
18	2	100%	15.81–100%	2	100%	15.81–100%
22	1	100%	2.5–100%	1	100%	2.5–100%

There were total 144 AEs (96 non-serious and 48 serious) reported during the study, of which 15 were related and 129 were unrelated to rituximab administration according to the investigators. Out of the 15 related AEs 11 were non-serious and 4 were serious (cardiac failure, febrile neutropenia). All AEs related to rituximab administration were resolved during the course of the study. Overall, 7 patients died because of SAEs unrelated to rituximab. The most common adverse event occurred in connection with the administration of rituximab was infusion related reaction. In general, no additional safety data relevant for the therapeutic use of rituximab were identified.

## Discussion

This non-interventional study was primary designed to investigate the efficacy of rituximab containing chemotherapeutic regimens in previously untreated CLL. The predominance of male patients as well as the median age of the study population are in line with the known epidemiological characteristics of the disease. At the time of enrollment, the majority of patients had advanced stage disease, as reflected by Binet scores. It is though noteworthy, that approximately 10% of the ITT population was ranked to have Binet stage A. At this stage, it is rather unconventional to commence therapy, though it is evident that treating physicians did not solely rely on this staging system when determining disease progression. This may also relate to the apparent lack of well-defined and accepted criteria for the progression of CLL.

Furthermore, a considerable heterogeneity regarding the applied concomitant chemotherapeutic regimens has also been observed. Analysis of study data showed, that in a real-life setting, those first line protocols were administered most frequently (FCR, R-bendamustin, R-chlorambucil), that are generally considered as the standard first-line therapies by relevant clinical guidelines [17]. It should be mentioned that FCR was frequently chosen for the fit older patients as the access to bendamustin was limited at that time. Nevertheless, other non-standard therapeutic approaches (such as R-CVP, or even R-CHOP, R-CAP) are also apparent from this study. This finding could be explained with the fact, that until recently R-CVP was commonly used in the treatment of CLL, thus there is a wide experience regarding its use and safety.

Due to major limitations arising from the single arm design of this study, overall response rates evaluated as primary and secondary endpoints can be interpreted only in the context of published clinical data provided by previous clinical investigations. Several Phase II and Phase III trials investigated the efficacy of rituximab administered in combination with different chemotherapeutic regimens to previously untreated CLL patients. When combined with fludarabine, rituximab treatment resulted in ORRs of 87% [21] and 90% [10]. In a more recent Phase II trial an ORR of 88% was calculated for rituximab-bendamustine combination [22]. Regarding the FCR regimen, an early trial indicated an ORR as high as 95% (CI_95%_: 92%–98%) [23], though later a larger randomized Phase III trial reported a somewhat lower ORR of 90% [24]. Low dose FC in combination with high dose rituximab (FCR-Lite) also resulted in 88% ORR [25]. These previously reported results are in tune with the ORR observed in the overall population of this study.

Analysis of response rates in different age groups revealed a slightly higher ORR in patients aged at or above 65 years. Similar observation was made by Hallek et al. [24] as these authors have found ORR of 93 and 89% in age groups of ≥65 years and < 65 years, respectively. Interpretation of response rates calculated on the basis of either chromosomal mutation status or comorbidity-indicating CIRS scores, however, meets common difficulties. Although ORR appeared to be as high as 100% in several subgroups, the biasing effect of the low patient number is clearly reflected from the wide confidence intervals. The proportion of patients with either 17p or 11q deletions were lower compared to those reported in previous clinical studies [26]. The fact that only the 60% of the ITT population was evaluated by FISH further reveals evident gaps in the routine clinical practice in Hungary. It should be noted that since the availability of ibrutinib, FISH is required before every new line of treatment according to local recommendation. FISH test is available in the big academic centers only, but access to these tests has improved by sample transport. Program to assess TP53 mutation was also launched recently, it became available in three academic centers, one of them has already been accredited by the laboratory of the European Research Initiative on CLL (ERIC). Testing for the IgHV mutation status is always recommended by the iwCLL2018 guideline, which is a big change in consequence of recent long term results of several chemoimmunotherapy trials and the approval of ibrutinib in every line of treatment. Though direct comparison of sequential chemoimmunotherapy followed by BCRi and first line BCRi is lacking, first line ibrutinib is widely used in several countries, mainly for patients with unmutated IgHV. In Hungary, IgHV test became available routinely in the big academic centers, and one of them also ERIC accredited. Obtaining IgHV mutation status was not recommended in the period of this observational trial. Similarly, CIRS scores were provided 72 and 77% of the ITT and PP population, respectively, which indicates, that the complexity of CIRS score calculation limits its use in routine clinical practice. Nevertheless, the predictive role of chromosomal mutations and CIRS score cannot be confirmed upon these results.

In light of the data provided by earlier clinical studies, the results of the CILI study can be considered as being supportive for the efficacy and safety of the of rituximab used in combination with standard chemotherapy in previously untreated CLL patients. Rituximab therapy was well tolerated and there were no unexpected safety findings.
